# Competing endogenous RNA (ceRNA) hypothetic model based on comprehensive analysis of long non-coding RNA expression in lung adenocarcinoma

**DOI:** 10.7717/peerj.8024

**Published:** 2019-11-07

**Authors:** Xiwen Wang, Rui Su, Qiqiang Guo, Jia Liu, Banlai Ruan, Guiling Wang

**Affiliations:** 1 Department of The First Thoracic Surgery, Shengjing Hospital, China Medical University, Shenyang, Liaoning, China; 2 Institute of Translational Medicine, China Medical University, Shenyang, Liaoning, China; 3 Department of Cell Biology, Key Laboratory of Cell Biology, Ministry of Public Health and Key Laboratory of Medical Cell Biology, Ministry of Education, China Medical University, Shenyang, Liaoning, China; 4 College of Life Science, China Medical University, Shenyang, Liaoning, China

**Keywords:** Lung adenocarcinoma, TCGA, LncRNAs, ceRNA

## Abstract

**Background:**

Non-small cell lung cancer (NSCLC) is a major subtype of lung cancer with high malignancy and bad prognosis, consisted of lung adenocarcinomas (LUAD) and lung squamous cell carcinomas (LUSC) chiefly. Multiple studies have indicated that competing endogenous RNA (ceRNA) network centered long noncoding RNAs (lncRNAs) can regulate gene expression and the progression of various cancers. However, the research about lncRNAs-mediated ceRNA network in LUAD is still lacking.

**Methods:**

In this study, we analyzed the RNA-seq database from The Cancer Genome Atlas (TCGA) and obtained dysregulated lncRNAs in NSCLC, then further identified survival associated lncRNAs through Kaplan–Meier analysis. Quantitative real time PCR (qRT-PCR) was performed to confirm their expression in LUAD tissues and cell lines. The ceRNA networks were constructed based on DIANA-TarBase and TargetScan databases and visualized with OmicShare tools. Gene Ontology (GO) and Kyoto Encyclopedia of Genes and Genomes (KEGG) pathway analyses were performed to investigate the potential function of ceRNA networks.

**Results:**

In total, 1,437 and 1,699 lncRNAs were found to be up-regulated in LUAD and LUSC respectively with 895 lncRNAs overlapping (|log2FC| > 3, adjusted *P* value <0.01). Among which, 222 lncRNAs and 46 lncRNAs were associated with the overall survival (OS) of LUAD and LUSC, and 18 out of 222 up-regulated lncRNAs were found to have inverse correlation with LUAD patients’ OS (|log2FC| > 3, adjusted *P* value < 0.02). We selected 3 lncRNAs (CASC8, LINC01842 and VPS9D1-AS1) out of these 18 lncRNAs and confirmed their overexpression in lung cancer tissues and cells. CeRNA networks were further constructed centered CASC8, LINC01842 and VPS9D1-AS1 with 3 miRNAs and 100 mRNAs included respectively.

**Conclusion:**

Through comprehensively analyses of TCGA, our study identified specific lncRNAs as candidate diagnostic and prognostic biomarkers for LUAD. The novel ceRNA network we created provided more insights into the regulatory mechanisms underlying LUAD.

## Introduction

Lung cancer is one of the most frequently diagnosed cancers and leading causes of cancer-related mortality worldwide with nearly 1.8 million new cases every year (Ferlay et al., 2015). Lung cancer is often diagnosed at an advanced stage of disease with a 5-year age-standardized survival lower than 20% (Carozzi et al., 2017). As the predominant type, non-small cell lung cancer (NSCLC) accounts for approximately 85–90% of lung cancer cases (Li et al., 2017). Lung adenocarcinoma (LUAD) is the most common pathologic subtype of NSCLC, which also comprises nearly 40% of lung cancer cases (Xiong et al., 2018). Although various advances have been made in diagnosis and clinical treatment, the overall survival (OS) time and recurrence rate of LUAD patients remain dismal (Lin et al., 2016). Thus, disclosing the underlying molecular mechanisms and investigating possible diagnostic and therapeutic targets of LUAD should be emphasized.

The impact of genomic aberrations on protein is known as a risk factor that associated with complex diseases including cancer (Califano et al., 2012). In addition to protein-coding gene mutations, non-coding RNAs (ncRNAs) dysregulation also plays an established role in tumorigenesis (Chiu et al., 2017). Traditionally believed to represent transcriptional noise, ncRNAs account for nearly 98% of human genome, have recently been suggested to possess functional roles in transcriptional, post-transcriptional and epigenetic processes (Sana et al., 2012). Long non-coding RNAs (lncRNAs) and microRNAs (miRNAs) are the two most common subtypes of ncRNAs (Viereck & Thum, 2017). MiRNAs are a group of short single-stranded ncRNAs with about 22 nucleotides long, can function as regulators by binding to 3′ untranslated regions (3′ UTRs) of their target mRNA transcripts. The binding of miRNAs on mRNAs can cause either mRNAs degradation or mRNAs translational inhibition (McGuire, Brown & Kerin, 2015). Emerging evidence has indicated that miRNAs can act as key regulators in cancer by linking related RNAs into their complex networks of interactions (Anastasiadou, Jacob & Slack, 2018). Among them, competitive endogenous RNA (ceRNA) hypothesis is about a regulatory mechanism between ncRNAs and coding RNAs, which postulates that RNA transcripts can sequester miRNAs from other targets by sharing the same MREs (Salmena et al., 2011). Specifically, the increase (or decrease) of possible ceRNAs (such as lncRNAs and circular RNAs) can act as molecular sponges, attract (or release) miRNAs through MREs, thereby de-repressing (or degrading) the target mRNAs of respective miRNAs (Chiu et al., 2015). Recently, multiple reports found that the disturbed of ceRNA network plays important roles in the pathogenesis of cancers that include LUAD (Kumar et al., 2015; Cong et al., 2019).

Numerous species of RNAs can serve as potential ceRNAs due to their MREs theoretically. Recently, several researchers dedicated to the prediction of putative ceRNA regulatory networks and their potential roles in cancers using bioinformatic methods (Zhang et al., 2018; Chiu et al., 2015). The Cancer Genome Atlas (TCGA) is a public database that contains both clinical and molecular data of thousands of tumor samples on over 33 different types (Colaprico et al., 2016). By using TCGA, a number of tumors-specific expressed genes and pathways in NSCLC were acquired by Li et al. (2018). Based on these genes, they performed downstream analyses including functional enrichment analysis, protein interaction analysis, survival analysis and further built a ceRNA network covers 124 dysregulated lncRNAs (Li et al., 2018). Similarly, Ning et al. (2018) constructed a ceRNA network and demonstrated that several lung squamous cell carcinomas (LUSC)-specific ceRNAs (PLAU, miR-31-5p, miR-455-3p, FAM83A-AS1, MIR31HG, MIR99AHG) are associated with the OS of LUSC patients. In LUAD, although numerous anomalous lncRNAs have been investigated studies with large scale samples size remain scarce.

Here, we investigated aberrantly expressed lncRNAs in NSCLC by analyzing tumor tissues and non-cancerous tissues from TCGA dataset. Among these dysregulated lncRNAs, we identified 18 lncRNAs that were up-regulated in NSCLC tissues and have inverse associations with the OS of LUAD patients. Furthermore, we selected 3 lncRNAs out of 18 (CASC8, LINC01842 and VPS9D1-AS1), and confirmed their overexpression via qRT-PCR. Importantly, we constructed a ceRNA network that centered on CASC8, LINC01842 and VPS9D1-AS1 in LUAD and analyzed the possible functions of these three lncRNAs in their ceRNA network by conducting functional annotation analyses respectively. This study aimed to investigate specific lncRNAs and related ceRNA networks that may be involved in the molecular mechanisms of LUAD and provide potential diagnostic biomarkers for LUAD.

## Materials and Methods

### Data of patients and computational analysis

The lncRNA expression profiles and clinical information of LUAD and LUSC were downloaded from TCGA database. In total, 535 tumor tissues with 59 control samples of LUAD and 502 tumor tissues with 49 adjacent non-tumorous lung tissues of LUSC were collected in this analysis. The lncRNAs expression profile were transformed by ENSEMBL project to define and encode first (htps://www.ensembl.org/). Differentially expressed lncRNAs between tumor tissues versus normal tissues were identified using the package of DESeq2 in R language with |log2FC| > 2 and adjusted *P* value < 0.01 set as the thresholds. Based on the clinical data, the associations between lncRNAs’ expression and lung cancer patients’ OS time were performed by survival analysis. Thereafter, Kaplan–Meier survival analysis were carried out and visualized using “survival” package in R. The receiver operation characteristic (ROC) curves analysis along with the area under the ROC curve (AUC) were used to estimate the diagnostic values (sensitivity and specificity) of specific lncRNAs by using the OmicShare tools (http://www.omicshare.com/tools).

### Clinical specimens

All human specimens (LUAD tissues, LUSC tissues and normal lung tissues) were collected from patients who were undergoing surgery at the Department of Thoracic Surgery, Shengjing Hospital of China Medical University. The fresh specimens were frozen in liquid nitrogen immediately until further use. The written informed consents were obtained from all patients, and this study was approved by Research Ethics Board (2018PS170K) at the Shengjing Hospital of China Medical University.

### Cell culture

The human bronchial epithelial cell line (BEAS-2B) and human non-small lung cancer cell lines (A549, H460, H1299 and H292) were purchased from Shanghai Institutes of Biochemistry and Cell Biology, Chinese Academy of Sciences (Shanghai, China). BEAS-2B cells were grown in LHC-8 (Gibco, Carlsbad, CA, USA) medium containing 10% fetal bovine serum (FBS) (Life Technologies Corporation, Paisley, UK). A549, H460, H1299 and H292 cells were cultured in high RPMI-1640 medium (Gibco, Carlsbad, CA, USA) containing 10% FBS. All these cells were incubated at 37 °C in a humidified incubator with 5% CO_2_.

### RNA extraction and qRT-PCR

The total RNA was separated from the tissues and cells using Trizol reagent (Invitrogen, Carlsbad, CA, USA) and RNA concentration was determined by NanoDrop 2000 spectrometer (Thermo Fisher, Waltham, MA, USA). The cDNA was generated using HisScript™ QRT SuperMix for qPCR (Vazyme Biotech, Nanjing, China) according to the manufacturer’s instructions. QRT-PCR reactions were performed using ChamQ™ Universal SYBR qPCR Master Mix (Vazyme Biotech, Nanjing, China) on Agilent Technologies Stratagene Mx3000P (Santa Clara, CA, USA). Expression was normalized using the relative quantification (2^−ΔΔCt^) method.

### Construction of ceRNA network

The construction of ceRNA network was based on the ceRNA hypothesis that lncRNA and mRNA could co-regulate each other by sharing MREs. In our study, the target miRNAs of specific lncRNAs were predicted by DIANA-TarBase v.8 (http://carolina.imis.athena-innovation.gr/diana_tools/web/index.php?r=tarbasev8%2Findex/) (Karagkouni et al., 2018). Targetscan 7.2 (http://www.targetscan.org/) was performed to predict mRNAs targeted by miRNAs. Furthermore, the lncRNA-miRNA-mRNA ceRNA networks were constructed and visualized using OmicShare tools (www.omicshare.com/tools/Home/Soft/cytoscape).

### Functional enrichment analysis

To further evaluate the biological functions of lncRNA-associated ceRNA networks, Gene Ontology (GO) and Kyoto Encyclopedia of Genes and Genomes (KEGG) pathway enrichment analyses were performed using the OmicShare tools, a free online platform for data analysis (www.omicshare.com/tools). In GO analysis, all genes were first mapped to GO terms in the GO database (http://www.geneontology.org/), and gene numbers were calculated for each term. Significantly enriched GO terms and KEGG pathways were comparing with the whole genome background. The calculated *P*-value of both analyses were gone through the false discovery rate (FDR) Correction, taking FDR ≤ 0.05 as a threshold.

### Statistical analysis

All the data were presented as mean ± SD. The SPSS version 22.0 statistical software (IBM, NY, USA) and R language were used for data analysis with the Student’s *t*-test (two tailed) or one-way analysis of variance for multiple groups. Adjusted *P* values less than 0.05 were considered as statistically significant.

## Results

### Identification of lung cancer specific lncRNAs

**Figure 1:**
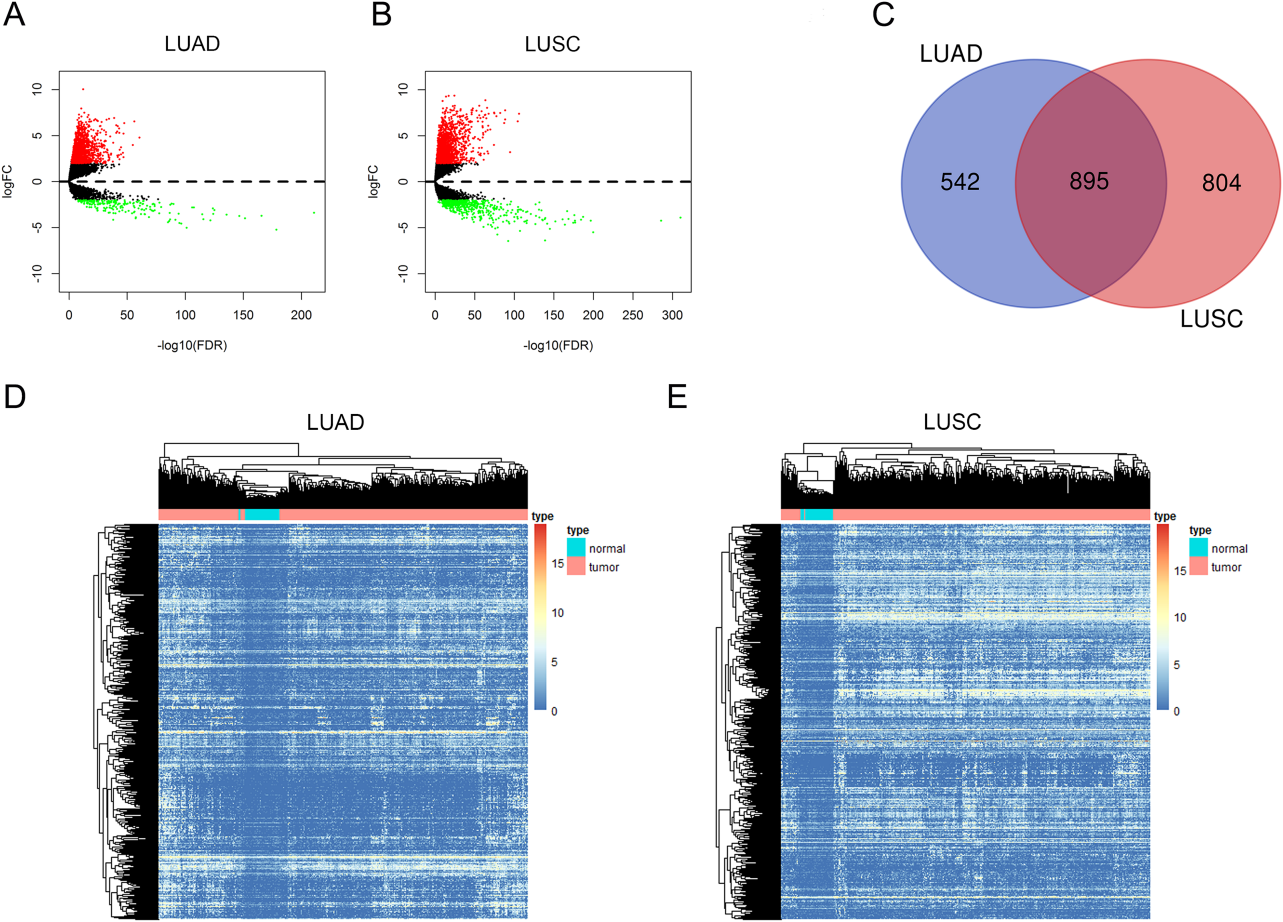
Aberrantly expressed lncRNAs in LUAD and LUSC. Volcano plot of DElncRNAs in LUAD (A) and in LUSC (B). The red dot symbolizes up-regulated lncRNAs and the green dot symbolizes down-regulated lncRNAs with absolute logFC > 2 and corrected *P* < 0.01 as thresholds. (C) Venn diagram shows 895 overlapping up-regulated lncRNAs in both LUAD and LUSC. Heat maps of the 895 up-regulated lncRNAs in LUAD (D) and in LUSC (E). The color from blue to red shows a trend from low expression to high.

In this study, we investigated the RNA expression levels in 535 LUAD samples compared with 59 adjacent non-tumorous tissues and 502 LUSC samples compared with 49 normal tissues, and the RNA-sequencing data were all obtained from TCGA database. We first identified dysregulated lncRNAs in LUAD and LUSC with the cutoff criteria as |log2FC| > 2 and adjusted *P* value < 0.01 (Figs. 1A and 1B, the details of dysregulated lncRNAs were list in Supplemental File 1). Among these dysregulated lncRNAs, a total of 1,437 and 1,699 lncRNAs were detected to be up-regulated in LUAD and LUSC tissues. A Venn diagram analysis and cluster analysis maps were used to represent 895 overlapping lncRNAs between up-regulated lncRNAs in LUAD and LUSC (Figs. 1C–1E, up-regulated lncRNAs were listed in Supplemental File 2).

To further identify the prognostic characteristics of 895 lncRNAs, survival analyses were applied. By conducting Kaplan–Meier curve analysis, 222 and 46 up-regulated lncRNAs were found to have associated with LUAD and LUSC patients’ OS respectively (log-rank, *P* < 0.05), while 85 out of 222 lncRNAs and 10 out of 46 lncRNAs were negatively correlated with patients’ OS. To further enhance the reliability, we screened data further that 18 out of 895 up-regulated lncRNAs were negatively correlated with patients’ OS in LUAD with |log2FC| > 2 and adjusted *P* value <0.02 as thresholds (Supplemental File 3). The results of survival analysis and ROC curve of 18 lncRNAs were shown in Fig. S1

**Figure 2:**
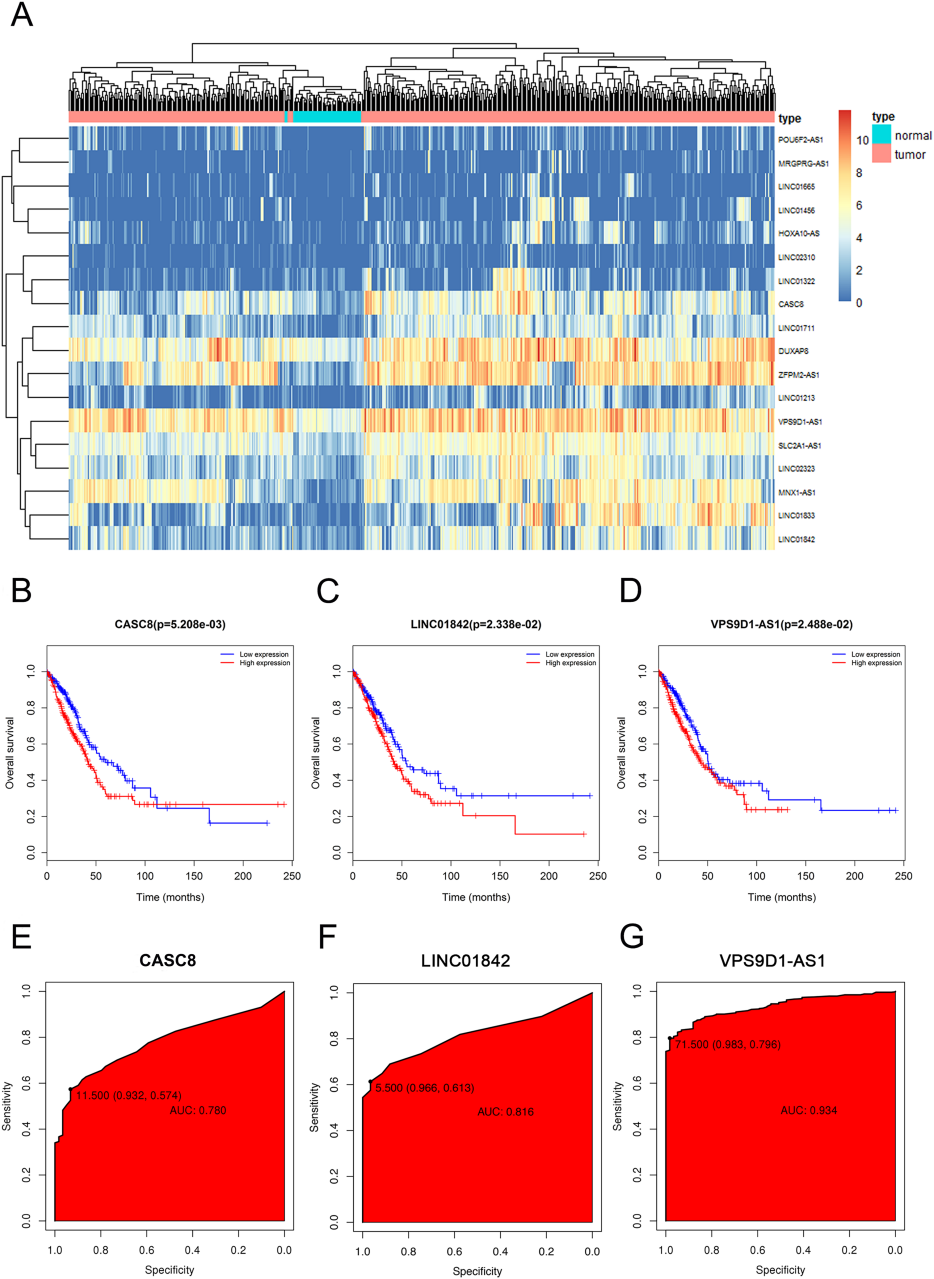
The elevated expression and prognostic characteristics of 3 selected lncRNAs. (A) Heatmap of upregulated expression of CACS8, LINC01842 and VPS9D1-AS1 in 535 LUAD tissues compared with 59 normal tissues according to TCGA dataset. Each column stands for one tissue and each row stands for one lncRNA. The Kaplan–Meier survival curves of CASC8 (B), LINC01842 (C) and VPS9D1-AS1 (D). The ROC curves of CASC8 (E), LINC01842 (F) and VPS9D1-AS1 (G).

In order to visualize the most specific lncRNAs, we depicted the expression of 18 lncRNAs in 535 LUAD tissues and 59 normal lung tissues that we downloaded (Fig. 2A). Furthermore, after discarding the lncRNAs which were poorly expressed in normal lung tissues, we selected three significant lncRNAs (CASC8, LINC01842, VPS9D1-AS1) for the subsequent study. As shown in Figs. 2B–2G, the elevated expressions of CASC8, LINC01842 and VPS9D1-AS1 indicated poorer prognosis of LUAD than low expression, the AUC of them three were 0.729, 0.821 and 0.954, respectively.

**Figure 3:**
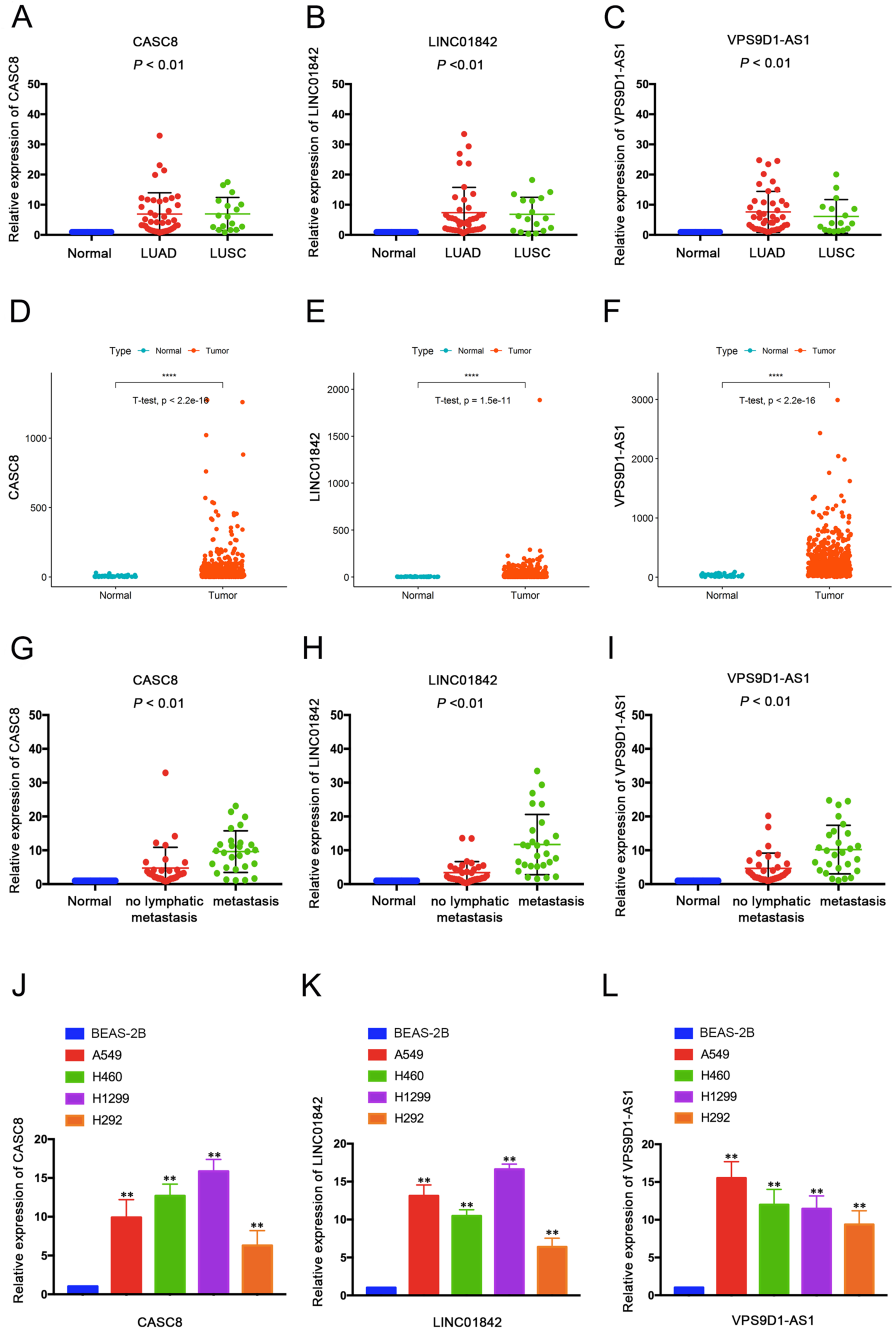
QRT-PCR validated three dysregulated lncRNAs in NSCLC tissues and cells. Expressions of CACS8 (A), LINC01842 (B) and VPS9D1-AS1 (C) were significantly elevated in LUAD tissues (*n* = 42) and LUSC tissues (*n* = 17) compared with adjacent non-tumorous lung tissues. The up-regulated expressions of CACS8 (D), LINC01842 (E) and VPS9D1-AS1 (F) in LUAD tissues (*n* = 535) compared with normal lung tissues (*n* = 59) were validated based on the TCGA database. QRT-PCR results indicated that the elevated expression levels of CACS8 (G), LINC01842 (H) and VPS9D1-AS1 (I) were correlated with lymph metastasis (*n* = 27, with lymphatic node metastasis; *n* = 32, without). CACS8 (J), LINC01842 (K) and VPS9D1-AS1 (L) were up-regulated in NSCLC cell lines A549, H460, H1299 and H292 compared with normal lung cells BEAS-2B (*n* = 5, each group). Data are presented as mean ± SD. **P < 0.01.

Thereafter, we validated the expression data of 3 selected lncRNAs using quantitative real-time PCR (qRT-PCR) between 59 NSCLC tissues and their corresponding normal tissues. The detailed clinical and pathological features of 59 samples are shown in Supplemental File 4. As qRT-PCR results shown (Figs. 3A–3C), CASC8, LINC01842 and VPS9D1-AS1 were significantly up-regulated in LUAD and LUSC tissues and the results were consistent with TCGA dataset (Figs. 3D–3F). Moreover, we found that the expression level of the three lncRNAs were significantly elevated in the patients with lymph node metastasis compared with the patients without (Figs. 3G–3I). In addition, qRT-PCR indicated their elevated expression in NSCLC cell lines (A549, H460, H1299 and H292) compared with normal lung cells BEAS-2B (Figs. 3J–3L).

### Construction of ceRNA network

**Figure 4:**
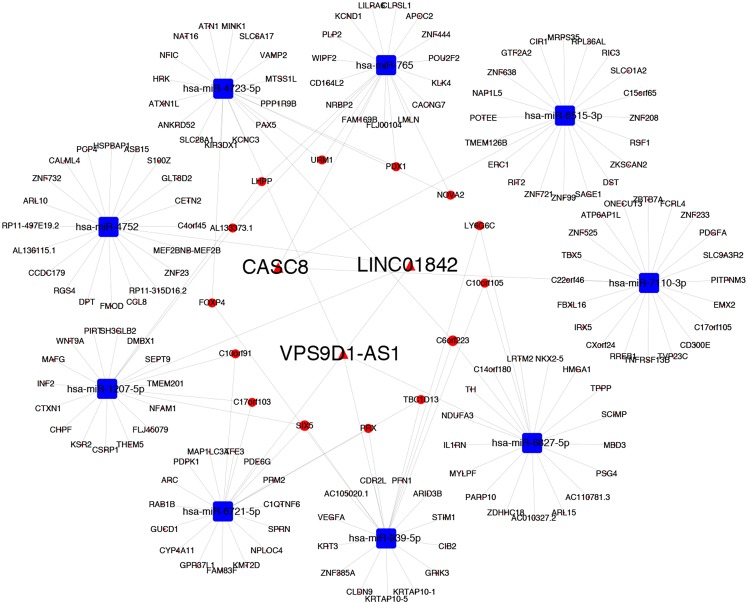
CeRNA network centered on CACS8, LINC01842 and VPS9D1-AS1 in LUAD.

To further explore the underlying molecular mechanisms of CASC8, LINC01842 and VPS9D1-AS1 in LUAD, three lncRNA-related ceRNA networks were constructed. Based on the prediction using DIANA-TarBase v.8 (Karagkouni et al., 2018), three potential binding miRNAs that with top binding scores were selected for CASC8 (miR-6515-3p, miR-7110-3p, miR-765), LINC01842 (miR-4752, miR-6721-5p, miR-1207-5p) and VPS9D1-AS1 (miR-6827-5p, miR-939-5p, miR-4723-5p). Then the target 100 mRNA genes of these miRNAs were searched using TargetScan. Three networks were presented in Fig. S2, the miRNA-target mRNAs were list in Supplemental File 5. Finally, after combining the interactions between lncRNAs-miRNAs and miRNAs-mRNAs, we used OmicShare to visualize the network of top target 20 mRNAs (Fig. 4). Further analysis of the network revealed that it contained 192 nodes and 378 edges.

### Function annotation of key DElncRNAs in the ceRNA network

**Figure 5:**
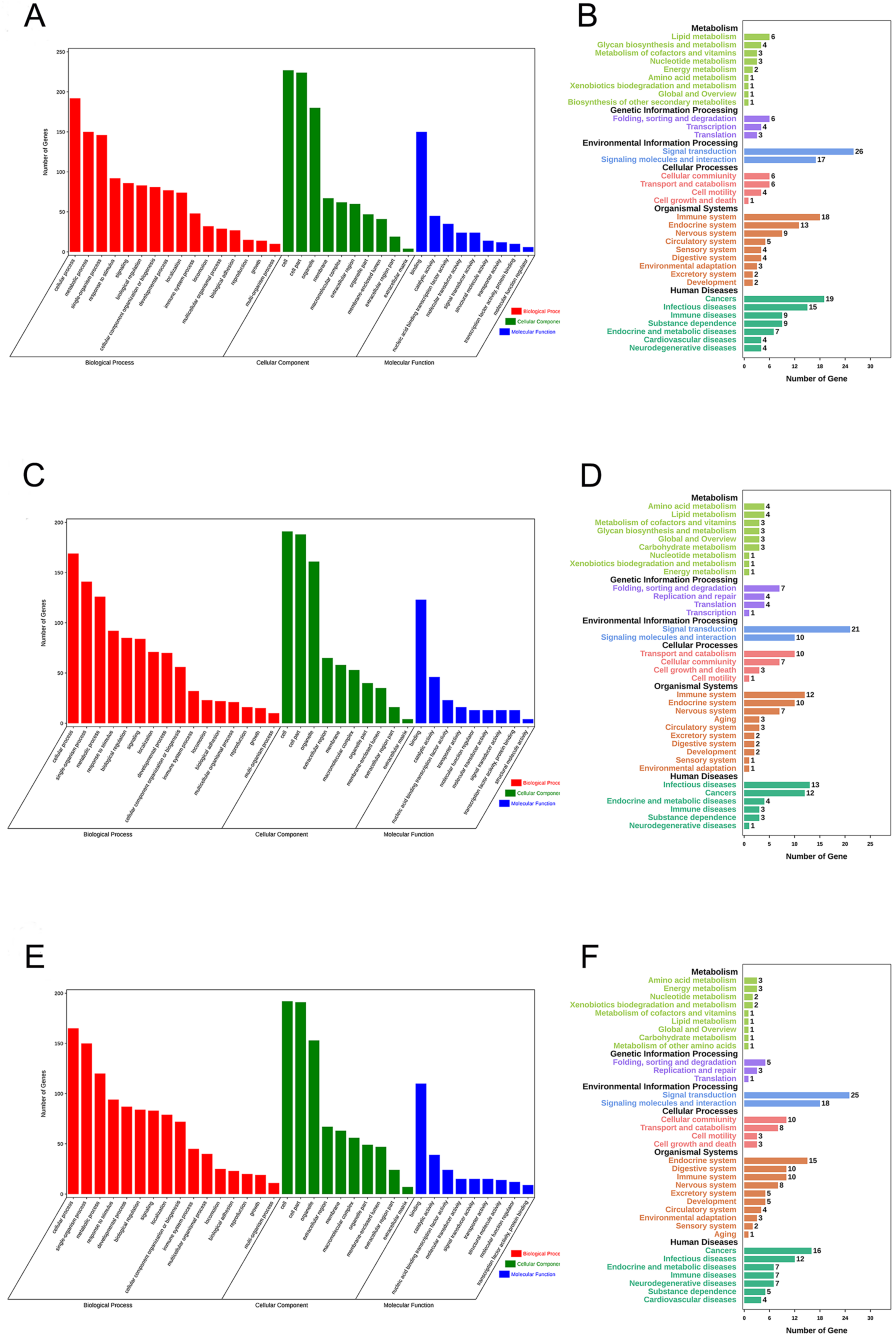
Functional enrichment analyses for mRNAs in three ceRNA networks. GO analysis and KEGG pathways analysis for mRNAs that involved in CASC8-related ceRNA network (A and B), LINC01842-related ceRNA network (C and D) and VPS9D1-AS1-related ceRNA network (E and F) respectively. Analyses were performed using OmicShare tools. The calculated *P* values were gone through FDR correction, taking FDR ≤ 0.05 as thresholds.

Gene Ontology (GO) enrichment analysis and KEGG pathway analysis were performed to assess the potential biological functions of CASC8, LINC01842 and VPS9D1-AS1. Based on their lncRNA-miRNA-mRNA ceRNA network, we analyzed the mRNAs involved. The basic unit of GO is GO-term, each GO-term belongs to a type of ontology. GO enrichment analysis provided all GO-terms that significantly enriched in mRNAs comparing to the genome background. Thus, the top 16 GO biological process terms, top 10 GO cellular component terms and top nine GO molecular function terms were presented in Fig. 5. We also analyzed enriched metabolic pathways or signal transduction pathways in mRNAs involved using KEGG pathway analysis. A total of 35 pathways in six cancer-related subgroups were significantly enriched in our results (Figs. 5A–5F). Analyses took FDR ≤ 0.05 as threshold.

## Discussion

Lung cancer, a leading cause of cancer death worldwide, consists of small cell lung cancer and NSCLC. NSCLC accounts for approximately 90% of lung cancer, and is further subdivided into three categories: LUAD, LUSC and large cell carcinomas (LCC), with LUAD and LUSC are the two main subclasses (Campbell et al., 2016). The majority of NSCLC patients are diagnosed with locally advanced, tumor recurrence or metastatic disease, leading to a high mortality and limited treatments (Socinski et al., 2016). Recently, numerous researchers are focusing on exploring biomarkers that could provide potentially effective target treatments (Vargas & Harris, 2016).

Long noncoding RNAs (lncRNAs) are a cluster of noncoding transcripts greater than 200 nucleotides in size. Up to now, an increasing amount of dysregulated lncRNAs have been identified to play vital roles in human cancers including NSCLC. For example, up-regulated expression of LINC00963 promoted migration and invasion of NSCLC cells and correlated with poor prognosis of NSCLC patients (Yu et al., 2017). Chen et al. (2016) identified LINC00473 as an effective target to block NSCLC growth for its necessity in maintaining NSCLC cell growth and survival.

In the present study, we analyzed dysregulated lncRNAs between NSCLC tissues and adjacent non-tumorous tissues by using the RNA-seq dataset from TCGA. A total of 1,437 lncRNAs and 1,699 lncRNAs were found to be up-regulated in LUAD and LUSC, and 895 lncRNAs overlapping. We next focused on the effects of these 895 lncRNAs on OS, and identified 222 and 46 survival-related lncRNAs in LUAD and LUSC based on the clinical data. Then, we excluded the genes that were not annotated and obtained 18 up-regulated lncRNAs that were negatively correlated with LUAD patients’ OS with |log2FC| > 3 and adjusted *P* value < 0.02 as thresholds. Among these 18 LUAD-specific lncRNAs, three lncRNAs (CASC8, LINC01842, VPS9D1-AS1) were selected for further verification by using TCGA data and qRT-PCR orderly. We validated the expression of CASC8, LINC01842 and VPS9D1-AS1 in the tissues of 59 LUAD patients and LUAD cell lines using qRT-PCR, and our results were consistent with the expression data from TCGA. Interestingly, cancer susceptibility candidate eight (CASC8) is a lncRNA that has been reported to be involved in various of cancers by its gene polymorphism (Yeager et al., 2007; Ma et al., 2015), including lung cancer (Hu et al., 2016). Moreover, consistent with our study, Tan & Yang (2018) identified VPS9D1-AS1 as an up-regulated lncRNA in NSCLC tissues using RNA-scope in situ hybridization, and further demonstrated that the expression levels were correlated with the prognosis of NSCLC patients.

Salmena et al. (2011) proposed a new network in post transcriptional regulation named the ceRNA hypothesis in 2011. This theory suggested that RNA transcripts could regulate the expression of each other by competitively sharing common miRNA response elements (MREs). Numerous studies have found that lncRNAs can act as ceRNAs by sequestering miRNAs from their mRNA targets, thereby regulate the expression of target mRNAs (Barbagallo et al., 2018; Gao et al., 2018). This lncRNA-miRNA-mRNA model has been reported as a new regulatory mechanism that functions in multiple biological processes including cancers (Shen et al., 2018; Witkos et al., 2018; Chen et al., 2017). In NSCLC, it was reported that lncRNA-SNHG7 functioned as a molecular sponge and sequestered miR-193b from FAIM2, thereby impaired miR-193b/FAIM2-induced tumor progression (She et al., 2018). Besides, lncRNA-NEAT1 promoted malignant biological behaviors of LUAD cells through sponging miR-193a-3p and abrogated the suppression of miR-193a-3p on USF1 (Xiong et al., 2018). Moreover, based on sequencing profiles from TCGA database, Sui et al. (2016) identified LUAD specific lncRNAs that correlated with clinical features and further constructed an lncRNA-miRNA-mRNA ceRNA regulatory network in LUAD via starBase.

In our study, we recognized three LUAD specific lncRNAs: CASC8, LINC01842 and VPS9D1-AS1 as key lncRNAs, and believed that there may be lncRNA-miRNA-mRNA cross-talks within these lncRNAs. First, we predicted the interaction between key lncRNAs and miRNAs using DIANA-TarBase v.8, and selected three miRNAs with top binding scores for each lncRNA respectively. MiR-6515-3p, miR-7110-3p and miR-765 for CASC8, miR-4752, miR-6721-5p, miR-1207-5p for LINC01842 and miR-6827-5p, miR-939-5p, miR-4723-5p for VPS9D1-AS1. Second, 100 target mRNAs of these miRNAs were predicted by Targetscan 7.2. Then, we used OmicShare to construct and visualize the ceRNA networks that focused on CASC8, LINC01842 and VPS9D1-AS1 respectively and together. The constructed network hypothesizes how these lncRNAs synergistically regulate the progression of NSCLC by co-regulation of multiple miRNAs and mRNAs. Specifically, many overlapped mRNAs among these three ceRNAs were reported to be oncogenes in various cancers, which are consist with our study. For instance, PDX1 was involved in both CASC8-related and VPS9D1-related networks, and also recognized as a key regulator and a potential target in pancreatic cancer (Wu et al., 2014). In VPS9D1-related networks that we built, FOXP4 is a common target of miR-6827-5p, miR-939-5p and miR-4723-5p. Moreover, FOXP4 is claimed to be a critical regulator in NSCLC and renal cacinoma (Yang et al., 2015; Xiong, Zhang & Song, 2019). SIX5 is participated in both ceRNA networks of LINC01842 and VPS9D1-AS1 and reported to overexpressed in NSCLC tissues and correlated with the tumor grade (Liu et al., 2016).

Finally, to understand more about the underlying biological processes involved in ceRNA networks that we built, we investigated the top enriched functional annotation of GO and KEGG pathway. GO is an international standardized gene functional classification system. As a result, these mRNAs involved were significantly enriched in 35 GO terms and 34 pathways. These significant GO terms were mainly involved in cellular process, metabolic process and single-organism process terms in biological process, cell, cell part and organelle terms in cellular component, and binding in molecular function. KEGG is the major public pathway-related database and able to identify significantly enriched metabolic pathways or signal transduction pathways in mRNAs. The pathway analysis results showed primarily pathways involved in these three ceRNA networks are signal transduction, signaling molecules and interaction, immune system, infectious disease and cancers.

In summary, based on the TCGA database, we investigated dysregulated lncRNAs in lung cancer, identified 18 lncRNA that were up-regulated and related to the OS time of LUAD patients. Then, we confirmed the elevated expression of CASC8, LINC01842 and VPS9D1-AS1 out of 18 lncRNAs in 59 pairs LUAD patients using qRT-PCR and TCGA analysis. Moreover, we constructed three lncRNA-miRNA-mRNA ceRNA networks of CASC8, LINC01842 and VPS9D1-AS1, respectively. Our findings provide potential therapeutic targets for LUAD and the ceRNAs that we constructed reveal unknown ceRNA regulatory networks in LUAD.

## Supplemental Information

10.7717/peerj.8024/supp-1 Supplemental Information 1 Kaplan–Meier curves for 18 up-regulated lncRNAs with LUAD patients’ OS. Survival analyses results of 18 lncRNAs [LINC01833 (A), LINC01456 (B), HOXA10-AS (C), ZFPM2-AS1 (D), MNX1-AS1 (E), CASC8 (F), LINC01322 (G), LINC02310 (H), LINC01842 (I), POU6F2-AS1 (J), LINC01665 (K), VPS9D1-AS1 (L), LINC01711 (M), LINC01213 (N), DUXAP8 (O), SLC2A1-AS1 (P), LINC02323 (Q) and MRGPRG-AS1 (R)]. (S) ROC analysis of 18 lncRNAs for diagnostic values in LUAD.

10.7717/peerj.8024/supp-2 Supplemental Information 2 Bioinformatic prediction of three lncRNA-miRNA-mRNA interactions centered on CACS8 (A), LINC01842 (B) and VPS9D1-AS1 (C), respectively. The ceRNA networks were constructed and visualized with OmicShare tools.

10.7717/peerj.8024/supp-3 Supplemental Information 3 The dysregulated lncRNAs in LUAD tissues (Table S1) and LUSC tissues (Table S2) compared with adjacent non-tumorous tissues by using the RNA-seq dataset from The Cancer Genome Atla. |log2FC| >2, adjusted *P* value <0.01.

10.7717/peerj.8024/supp-4 Supplemental Information 4 1437 lncRNAs were upregulated in LUAD (Table S3), 1699 lncRNAs were up-regulated in LUSC (Table S4) with 895 lncRNAs overlapping (Table S5).

10.7717/peerj.8024/supp-5 Supplemental Information 5 18 dysregulated lncRNAs were negatively correlated with LUAD patients’ OS. |log2FC| > 2, adjusted *P* value <0.02.

10.7717/peerj.8024/supp-6 Supplemental Information 6 The detailed clinical characteristics of 59 patients.

10.7717/peerj.8024/supp-7 Supplemental Information 7 Putative mRNA targets that may share MREs with CACS8 (Table S8), LINC01842 (Table S9) and VPS9D1-AS1 (Table S10), respectively.

## Additional Information and Declarations

### Data Availability

The following information was supplied regarding data availability: The raw measurements are available as Supplemental Files.
