# Supplementary figures and images for: Competing endogenous RNA (ceRNA) hypothetic model based on comprehensive analysis of long non-coding RNA expression in lung adenocarcinoma
Source: PeerJ. 2019 Nov 7;7:e8024. doi: 10.7717/peerj.8024 (PMC6842565; doi:10.7717/peerj.8024)

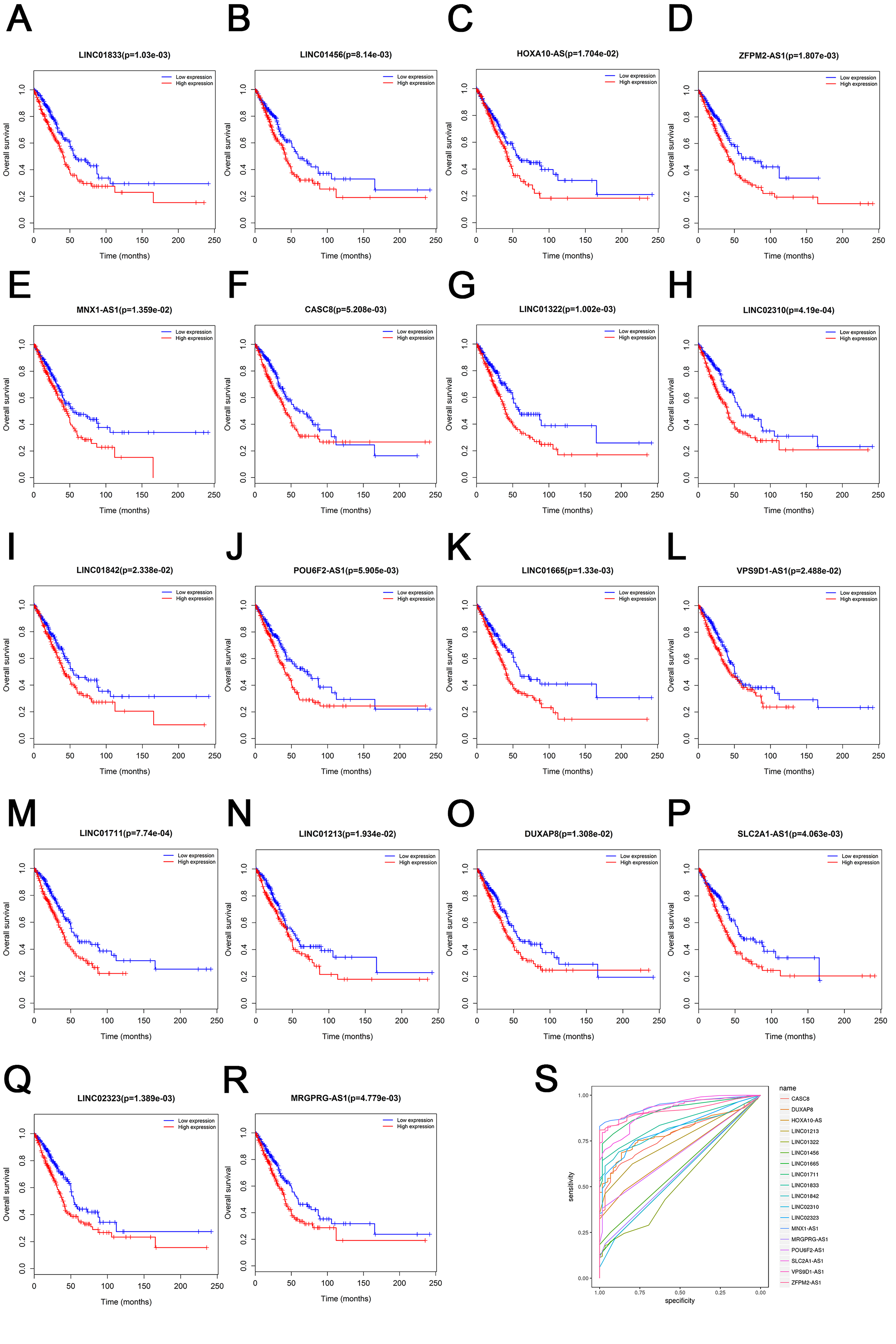

Supplement: Supplemental Information 1 — Survival analyses results of 18 lncRNAs [LINC01833 (A), LINC01456 (B), HOXA10-AS (C), ZFPM2-AS1 (D), MNX1-AS1 (E), CASC8 (F), LINC01322 (G), LINC02310 (H), LINC01842 (I), POU6F2-AS1 (J), LINC01665 (K), VPS9D1-AS1 (L), LINC01711 (M), LINC01213 (N), DUXAP8 (O), SLC2A1-AS1 (P), LINC02323 (Q) and MRGPRG-AS1 (R)]. (S) ROC analysis of 18 lncRNAs for diagnostic values in LUAD. [file peerj-07-8024-s001.png]
